# Effect of Multicomponent Training on Blood Pressure, Nitric Oxide, Redox Status, and Physical Fitness in Older Adult Women: Influence of Endothelial Nitric Oxide Synthase (NOS3) Haplotypes

**DOI:** 10.1155/2017/2578950

**Published:** 2017-09-14

**Authors:** Atila Alexandre Trapé, Elisangela Aparecida da Silva Lizzi, Thiago Correa Porto Gonçalves, Jhennyfer Aline Lima Rodrigues, Simone Sakagute Tavares, Riccardo Lacchini, Lucas Cezar Pinheiro, Graziele Cristina Ferreira, José Eduardo Tanus-Santos, Paula Payão Ovídio, Alceu Afonso Jordão, André Mourão Jacomini, Anderson Saranz Zago, Carlos Roberto Bueno Júnior

**Affiliations:** ^1^School of Nursing of Ribeirão Preto, University of São Paulo (USP), Avenida Bandeirantes 3900, 14049-900 Ribeirão Preto, SP, Brazil; ^2^Department of Mathematics, Federal University of Technology-Paraná (UTFPR), Avenida Alberto Carazzai, 1640, 86300-000 Cornélio Procópio, PR, Brazil; ^3^School of Medicine of Ribeirão Preto, University of São Paulo (USP), Avenida Bandeirantes 3900, 14049-900 Ribeirão Preto, SP, Brazil; ^4^School of Physical Education and Sport of Ribeirão Preto, University of São Paulo (USP), Avenida Bandeirantes 3900, 14049-900 Ribeirão Preto, SP, Brazil; ^5^Department of Physical Education, School of Science, São Paulo State University (UNESP), Avenida Engenheiro Luiz Edmundo Carrijo Coube, 14-01, Bairro Vargem Limpa, 17033-360 Bauru, SP, Brazil

## Abstract

The purpose of this study was to verify the influence of the genotype or haplotype (interaction) of the NOS3 polymorphisms [-786T>C, 894G>T (Glu298Asp), and intron 4b/a] on the response to multicomponent training (various capacities and motor skills) on blood pressure (BP), nitrite concentration, redox status, and physical fitness in older adult women. The sample consisted of 52 participants, who underwent body mass index and BP assessments. Physical fitness was evaluated by six-minute walk, elbow flexion, and sit and stand up tests. Plasma/blood samples were used to evaluate redox status, nitrite concentration, and genotyping. Associations were observed between isolated polymorphisms and the response of decreased systolic and diastolic BP and increased nitrite concentration and antioxidant activity. In the haplotype analysis, the group composed of ancestral alleles (H1) was the only one to present improvement in all variables studied (decrease in systolic and diastolic BP, improvement in nitrite concentration, redox status, and physical fitness), while the group composed of variant alleles (H8) only demonstrated improvement in some variables of redox status and physical fitness. These findings suggest that NOS3 polymorphisms and physical training are important interacting variables to consider in evaluating redox status, nitric oxide availability and production, and BP control.

## 1. Introduction

Population aging is occurring in all regions of the world; however, in recent years, it has been progressing more rapidly in developing countries [1]. By definition, human aging is defined as a dynamic and progressive process in which there are morphological, functional, biochemical, and psychological alterations, causing greater vulnerability and a greater incidence of pathological processes [2]. Among these changes, it is worth noting the decrease in physical fitness [3] and greater vulnerability to chronic diseases, especially cardiovascular diseases [4, 5].

These decreases in physical fitness over the aging process are directly associated with impairment in functional capacity [2]. It is important to emphasize that the regular practice of physical exercises can be considered as one of the main measures that counteract these factors. A multicomponent intervention seems to be appropriate [6] as the physical alterations related to the aging process affect various motor skills and abilities. The most recent official position of the American College of Sports Medicine (ACSM) [3, 7] provides indications for the practice of physical exercises, including training of the various capacities and motor skills (aerobic capacity, muscular strength, flexibility, coordination, agility, and balance).

Regarding the greater vulnerability to chronic diseases related to the aging process, there is an increase in the incidence of cardiovascular disease risk factors [5], with hypertension highlighted among the modifiable risk factors [4].

The etiology of hypertension is multifactorial and may involve genetic, environmental, and psychological aspects. Humoral factors are considered as a mechanism that controls blood pressure (BP), and certain alterations could result in elevation or a decrease in BP levels [8]. Among these, reduction in the production of nitric oxide (NO) may be related to aging, a sedentary lifestyle, and some genetic polymorphisms; besides, the reduction of bioavailability of NO may also be related to high exposure to oxidative stress. Reduced NO bioavailability is directly related to impairment in vasodilation, causing an increase in peripheral vascular resistance and raising BP values. There is a decrease in antioxidant activity, which may contribute to damage from exposure to oxidative stress [9–14]. High oxidative stress, a decrease in antioxidant activity, and low concentrations of NO are associated with increased BP values, especially in the context of the aging process [9, 11, 15].

It is known that endothelial cells are responsible for the synthesis, metabolism, and release of a wide variety of mediators that regulate vascular tone, with NO of paramount importance due to its role in BP control. NO production by nitric oxide synthase (NOS), which is made from the amino acid L-arginine, is responsive to physical exercise as shear stress in vessel walls promoted by blood flow is considered one of the most important physical stimuli for the endothelial cells to produce NO [9, 11, 15]. Therefore, improvement in physical fitness may increase NO concentration and decrease reactive oxygen species production. However, improvement in BP values is still controversial and different behavior can be found among individuals [15–17]. Although lifestyles with significant physical training and nutrition control have a huge influence on BP control, the genetic influence has been receiving special attention. Therefore, it is important to investigate the relationships and interactions between genetic polymorphisms, aging, and changes in lifestyle, such as the response to physical exercise [18, 19].

Among the many existing polymorphisms, some studies point out that genetic variants in the gene encoding nitric oxide synthase 3 (NOS3) -786T>C, 894G>T (Glu298Asp), and intron 4b/a may potentially explain the difference of declines characteristic of aging [19] and help to explain why some people demonstrate more benefits from physical training than others [18].

Some meta-analyses have shown the association between the NOS3 polymorphisms and hypertension, highlighting the intron 4b/a and 894G>T polymorphisms [20–22]. However, associations of NOS3 polymorphisms with hypertension and the response to physical training remain unclear, as well as the interaction among these polymorphisms, added to which, the previously mentioned study performed aerobic exercise.

Thus, the purpose of this study was to verify the influence of the genotype or haplotype (interaction) of the NOS3 polymorphisms [-786T>C, 894G>T (Glu298Asp), and intron 4b/a] on the response to multicomponent training for BP, nitrite concentration, oxidative stress, antioxidant activity, and physical fitness in older adult women. The hypotheses of the present study were that individuals carrying these NOS3 polymorphisms (the allele “C” of the gene -786T>C, the allele “a” of the gene intron 4, and GluAsp or AspAsp genotypes of the gene Glu298Asp) could present a worse response to physical training compared to ancestral carriers.

## 2. Methods

### 2.1. Ethical Review

This project was approved by the Ethics Committee of the Faculty of Philosophy, Sciences and Letters of Ribeirão Preto of the University of São Paulo (CAAE 24579513.4.0000.5407). All participants signed a free and informed consent form after having all questions answered by the researcher in charge and before starting the study. Although the present study only includes female participants that did not practice exercises in any other physical exercise program during the previous six months, male participants and individuals who were already training also participated in the interventions and evaluations, thus obtaining a return regarding health parameters. Only women were included in the analysis; this is justified by the fact that in physical training programs for the elderly, in general, there is a predominance of women, varying from 70 to 90% [14].

### 2.2. Study Design

**Figure 1:**
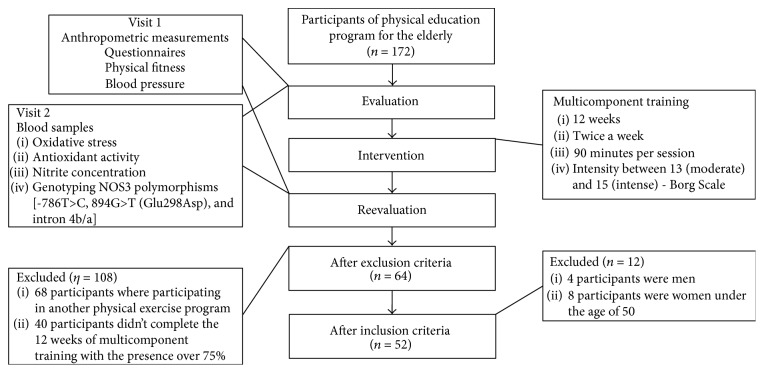
Flow chart: study design and sample selection.

The flow of participants and study design are illustrated in Figure 1.

#### 2.2.1. Sample Selection

All participants in the Physical Education program for the elderly, an extension project of the School of Physical Education and Sports of Ribeirão Preto (University of São Paulo), were invited to participate in this research. A convenience sample was obtained from this extension project. The subject's medical history was reviewed on their first visit. Inclusion criteria were being female and aged between 50 and 80 years. Exclusion criteria were the presence of any medical, mental, or musculoskeletal conditions that could prevent performance of the motor tests and physical training program; body mass index > 35 kg/m^2^, maximal systolic BP > 160 mmHg, and maximal diastolic BP > 100 mmHg; and participation in any other physical exercise program in six months prior to or during the intervention proposed by this study and presence <75% in the activities proposed by the intervention.

#### 2.2.2. Intervention and Evaluations

The duration of the intervention was 12 weeks, twice a week, on nonconsecutive days and each session lasted 90 minutes. All sessions were conducted by a physical education professional. The sessions were divided into four parts: (1) warm-up, including dynamic stretching exercises, coordination, and/or balance (about 20 to 30 minutes), (2) strength exercises performed in the form of a circuit using elastics, free weights, and body weight (about 30 to 40 minutes), (3) aerobic and ludic activities (dances or games) (about 20 to 30 minutes), and (4) “back to calm,” relaxation, massage, and stretching exercises (about 10 minutes). The intensity of the training was controlled by the Borg Scale [23], maintaining an intensity between 13 (moderate) and 15 (intense). Evaluations were performed before and after 12 weeks of intervention. The participants were submitted to the following assessments:

*(1) Anthropometric Measurements*. Body mass and height measurements were performed using a scale (graduation of 50 g) with a stadiometer (precision of 1.0 mm) (Welmy W200ALCD), which allowed calculation of the body mass index (BMI) using the equation weight/height^2^.

*(2) Questionnaires*. Level of physical activity was evaluated using the International Physical Activity Questionnaire (IPAQ) (interview that assesses frequency in days and duration in minutes of activities performed for more than ten minutes continuously in a normal week, classified as intense, moderate, and walking) [24] and assessment of nutritional status by the Food Consumption Marker Form (indicates the frequency of consumption of 10 food groups on the days of a regular week) [25]. The Brazilian Economic Classification Criterion is an instrument to estimate the purchasing power of urban families, leading to a classification of socioeconomic status. The scale presents a score that takes into account the possession and quantity of materials such as cars, refrigerators, and televisions, the schooling of the head of the family, and the presence of a domestic helper [26]. Although not included in the objectives, these three instruments were used to control for confounding factors.

*(3) Physical Fitness*. These tests are specific for older and elderly adults, validated and with normative reference values. Aerobic capacity was evaluated using the six-minute walk test (distance traveled on a rectangle route measuring 4.57 m × 18.28 m—the participant was required to walk as fast as possible, but without running), strength of the upper limbs was evaluated by the elbow and extension flexion test (the highest number of complete repetitions of elbow flexion and extension with the dominant arm in 30 seconds with the participant seated and using a dumbbell of 2.27 kg), and the lower limbs was evaluated by the sit and stand up test (the highest number of complete repetitions in 30 seconds of sitting and standing up from a chair—the participant was required to keep their arms crossed at the front of the trunk and touch the chair with the gluteus in each movement) [27].

*(4) Blood Pressure*. BP was measured using an automatic arm digital pressure gauge (OMRON brand, model HEM-7113), which uses the oscillometric measurement method. The measurement was performed at the first contact, with the participant remaining at rest for at least five minutes, according to the Brazilian Guidelines for Hypertension VII [4].

*(5) Blood Analysis*. Blood samples were drawn, and plasma was separated by 2000g centrifugation for 4 min at 24°C. Plasma samples were used for the analysis of oxidative stress, antioxidant activity, and nitrite concentration. Whole blood was used for the genotyping.

*(5.1) Nitrite (NO2) Concentrations*. Nitrite concentrations in plasma were used for the indirect determination of NO. Nitrite is the first product of the reaction of nitric oxide with oxygen. Plasma aliquots were analyzed in duplicate for their nitrite content using ozone-based chemiluminescence. Briefly, 300 *μ*l of plasma samples was injected into a solution of acidified tri-iodide, purging with nitrogen in line with a gas-phase chemiluminescence NO analyzer (Sievers Model 280 NO Analyzer, Sievers, USA). The data were analyzed using the Origin Lab 6.1 program [28].

*(5.2) Malondialdehyde (MDA)*. Malondialdehyde is one of the most abundant aldehydes resulting from tissue lipid peroxidation and can be considered a marker of global oxidative stress. In addition, it is related to the aging process [29]. This analysis was carried out according to the method proposed by Gerard-Monnier et al. [30], with some adaptations. For the determination of MDA in the plasma, 100 *μ*l of plasma was used. To this, 300 *μ*l of 10 mM solution of 1-methylphenylindole in acetonitrile and methanol (2 : 1, *v*/*v*) and 75 *μ*l HCl PA (37%) were added. Soon after, the tubes were vortexed and incubated in a water bath at 45°C for 40 minutes. After the bath, the samples were cooled on ice and then the tubes were centrifuged at 4000 rpm for 10 minutes. From the supernatant, absorbance was read in an apparatus (SpectraMax M3, Molecular Devices, USA) with a wavelength of 586 nm. The concentration of MDA was calculated using a hydrolyzed 1,1,3,3-tetramethoxypropane (TMP) curve [31].

*(5.3) Antioxidant Activity*. Antioxidant molecules prevent or inhibit the harmful reactions of reactive oxygen species. Plasma concentrations of different antioxidants can be measured in the laboratory separately, but the measurements are time-consuming, costly, labor-intensive, and often require complicated techniques. As the effect of these different antioxidants is additive, an alternative is to measure the total antioxidant capacity (TAC). This variable was measured using a method based on 2,2-azinobis 3-ethylbenzthiazoline-6-sulfonate (ABTS) by absorbance reading [32]. The other variable related to the antioxidant profile was glutathione (GSH), which plays a central role in the defense of cells against oxidative stress. This tripeptide is found intracellularly at high concentrations, primarily in all aerobic organisms. Glutathione is the most abundant low molecular weight cellular thiol. This analysis was performed using the method described by Costa et al. [33]. The concentration of glutathione was calculated using a reduced standard glutathione curve. Absorbance of these two experiments was also read in the same apparatus (SpectraMax M3, Molecular Devices, USA).

*(5.4) Genotyping*. The process of DNA extraction adopted was salting out [34]. The purity and DNA concentration of the sample were evaluated by spectrophotometry (BioDrop *μ*lite PC). The ratios 260/230 and 280/260 were evaluated, and the level of purity adopted as a satisfactory minimum was 1.7 for each of the ratios.

*(5.4.1) -786T>C*. The NOS3 polymorphisms at position -786T>C (rs2070744) were determined by real-time PCR (qPCR), as previously described. The reaction was carried out using Custom TaqMan allele discrimination assay (resynthesis part number AH5I790, Thermo Fisher, USA) and TaqMan genotyping master mix (Applied Biosystems, USA). Preparation of the reactions was performed according to the manufacturer's specifications for each sample: 1x of the master mix, 1x of TaqMan genotyping assay, and 50 ng of template DNA in 10 *μ*l final volume. Real-time PCR was performed on StepOnePlus equipment (Applied Biosystems, USA) and analyzed with the manufacturer's software [35].

*(5.4.2) 894G>T (Glu298Asp)*. Genotypes for the 894G>T (Glu298Asp) (rs1799983) polymorphisms were amplified by the PCR, as previously described, using the following flanking primers—sense: 5′-CATGAGGCTCAGCCCCAGAAC-3′ and antisense: 5′-AGTCAATCCCTTTGG TGCTCAC-3′. The amplicon was digested overnight at 37°C using 2 U *Mbo*I enzyme followed by electrophoresis for 3 h on 2.5% agarose gel. The G allele yields a fragment of 248 bp, and the T allele yields fragments of 190 and 58 bp [36].

*(5.4.3) Intron 4*. Genotypes for the variable number of tandem repeats (VNTR) polymorphism in intron 4 were determined by PCR, as previously described, using the primers 5′-AGG CCC TAT GGT AGT GCC TTT-3′ (sense) and 5′-TCT CTT AGT GCT GTG GTC AC-3′ (antisense) and fragment separation by electrophoresis for 3 h in 8% polyacrylamide gels. Fragments of 393 and 420 bp correspond to the endothelial nitric oxide synthase (eNOS) alleles 4a and 4b, respectively [37].

### 2.3. Statistical Analysis

The data analysis performed to achieve the proposed objectives for association and comparison among the interested variables was the Fisher's exact test to verify the statistical association of categorical variables with time (food intake) and linear mixed-effects models (random and fixed effects) adjusted for age, level of physical activity, and socioeconomic status. The classes of linear mixed-effects models are extensions of linear regression models for data collected and summarized in groups. These are used in the analysis of data in which the answers are grouped (repeated measures for the same individual); as we had information for the same individual in the pre and post time and the assumption of independence between observations in the same group is not adequate [38]. This model assumes that the residue obtained by means of the difference between the values predicted by the model and the observed values has a normal distribution with mean zero and constant variance. In these analyses, a level of significance of 5% was considered and the analysis was performed in SAS software (version 9.2) using the PROC MIXED.

## 3. Results

### 3.1. General Data

The mean age of the participants was 61.9 (8.7) (38.5% of the participants were between 50 and 59 years old and 61.5% were between 60 and 80 years old). Regarding the weekly frequency of intake of various foods [25], it was verified that there were no differences between the values before and after the multicomponent physical training intervention, showing that food habits remained similar during the intervention. In the same way, percentages of individuals using hypertension drugs were homogeneous among the groups for independent genotypes [-786: TT = 32% and TC + CC = 40.7%; Glu298Asp: GluGlu = 36.6% and GluAsp + AspAsp = 36.4%; and intron 4: bb = 34.3% and ba + aa = 41.2%] and haplotypes (interaction) [H1 = 33.33%; H2 = 25%; H3 = 33.33%; H6 = 30.76%; H7 = 44.44%; and H8 = 40%].

Table 1 summarizes the frequency distribution of NOS3 genotypes. The distribution of the genotypes of participants across groups indicates that no participant was classified as 4a4a for intron 4b/a. The distribution of genotypes for the three polymorphisms showed no deviation from Hardy-Weinberg equilibrium (*p* > 0.05) with allele frequencies of 0.70 and 0.30 for T and C allele at position -786T>C, respectively. The allele frequencies for G and 4b at position 894G>T (Glu298Asp) and intron 4b/a were 0.77 and 0.84, respectively. Correspondingly, the allele frequencies for T and 4a were 0.23 and 0.16, respectively.

Multicomponent training for 12 weeks decreased body mass [71.1 (11.2); 69.4 (10.9)—kg; *p* < 0.05] and BMI after the intervention compared to baseline [28.4 (4.7); 27.7 (4.8)—kg/m^2^; *p* < 0.05].  Table 2 shows that multicomponent training was effective in promoting a reduction in systolic and diastolic BP and malondialdehyde, as well as an increase in nitrite concentration, total antioxidant capacity, the flexion elbow test, sit and stand up test, and six-minute walking test. No changes were found in glutathione.

For the NOS3 genotype analysis, participants were divided into ancestral genotype groups [-786: TT (*n* = 25), Glu298Asp: GluGlu (*n* = 30), and intron 4: bb (*n* = 35)] and variant genotype groups [-786: TC + CC (*n* = 27), Glu298Asp: GluAsp + AspAsp (*n* = 22), and intron 4b/a: 4b4a + 4a4a (*n* = 17)] for each polymorphism.

### 3.2. Genotype Analyses

**Figure 2:**
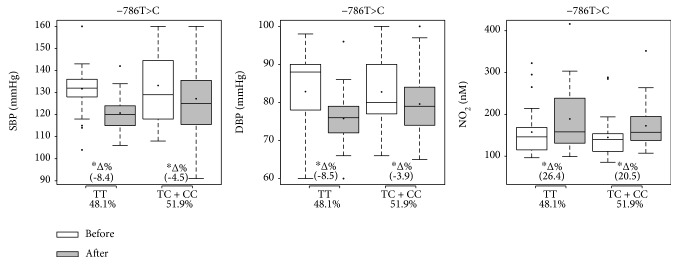
Effects of 12 weeks of multicomponent training on the systolic blood pressure (SBP), diastolic blood pressure (DBP), and nitrite concentration (NO_2_) of 52 older adult women with or without variant genotypes for the eNOS gene at position -786T>C. ^∗^*p* < 0.05 compared with before intervention (the same group). Linear mixed-effects models.

Figures 2(), 3(), and 4() present the effects of 12 weeks of multicomponent training on systolic and diastolic BP and nitrite concentration of participants for the NOS3 polymorphisms at positions -786T>C, Glu298Asp, and intron 4b/a, respectively. Variables did not differ between groups under basal conditions. Multicomponent training supported a statistical and significant reduction in both systolic and diastolic BP in all groups (-786T>C, Glu298Asp, and intron 4b/a), but the variant genotype groups descriptively presented trends towards a smaller magnitude of improvement (∆%) than the ancestral genotype groups. Similarly, all ancestral and variant genotype groups at position -786T>C and Glu298Asp presented improved nitrite concentration, but the variant genotype groups descriptively presented trends towards a smaller magnitude (∆%). In the intron 4b/a groups, 4b4b improved the nitrite concentration while multicomponent training did not affect this variable in the variant genotype group (4b4a+4a4a).

Tables 3, 4, and 5 show the effects of 12 weeks of multicomponent training on oxidative stress, antioxidant activity, and physical fitness of participants for the NOS3 polymorphisms at positions -786T>C, Glu298Asp, and intron 4b/a, respectively. MDA decreased in all groups (-786T>C, Glu298Asp, and intron 4b/a) in a similar fashion (ranging between 38.2 up to 51.8%). Regarding the antioxidant activity, all groups, with the exception of the 4b/a variant genotype group (4b4a+4a4a), increased total antioxidant capacity; for glutathione, only the ancestral genotype group showed improvement (-786: TT, Glu298Asp: GluGlu, and intron 4: 4b4b). Furthermore, 12 weeks of multicomponent training affected physical fitness in all groups (-786T>C, Glu298Asp, and intron 4b/a) which presented better results after the intervention in the elbow flexion, sit and stand up, and six-minute walking tests.

### 3.3. Haplotype Analysis

**Figure 5:**
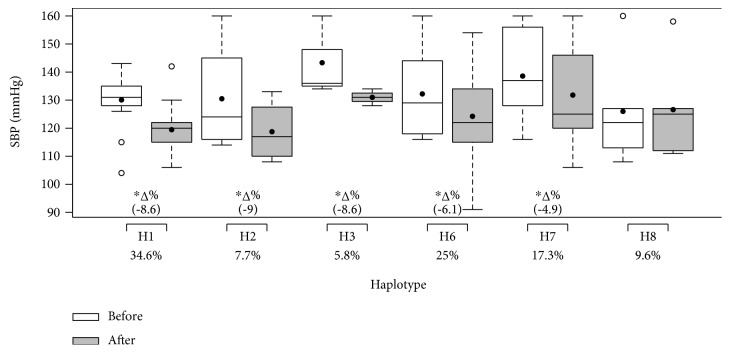
Effects of 12 weeks of multicomponent training on the systolic blood pressure (SBP) of 52 older adult women grouped by haplotype (H1–H8). ^∗^*p* < 0.05 compared with before intervention (the same group). Linear mixed-effects models.

**Figure 6:**
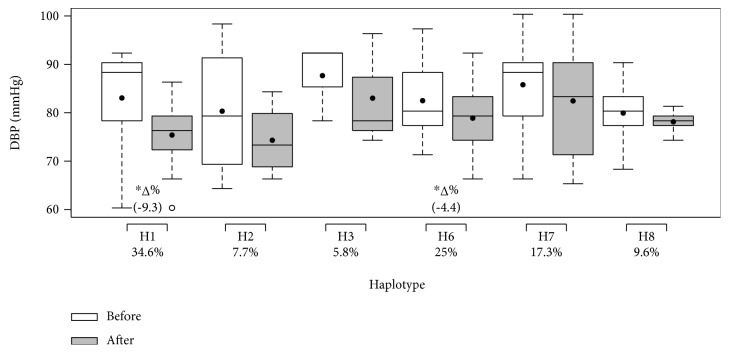
Effects of 12 weeks of multicomponent training on the diastolic blood pressure (DBP) of 52 older adult women grouped by haplotype (H1–H8). ^∗^*p* < 0.05 compared with before intervention (the same group). Linear mixed-effects models.

**Figure 7:**
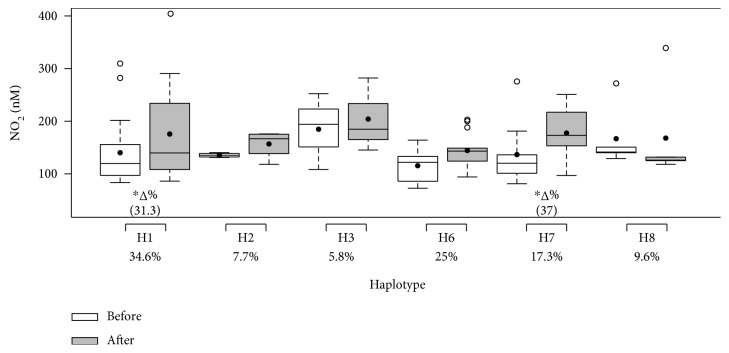
Effects of 12 weeks of multicomponent training on the nitrite concentration (NO_2_) of 52 older adult women grouped by haplotype (H1–H8). ^∗^*p* < 0.05 compared with before intervention (the same group). Linear mixed-effects models.

The haplotype frequencies and effects of 12 weeks of multicomponent training on the systolic and diastolic BP and nitrite concentration of participants grouped by haplotype (H1–H8) are illustrated in Figures 5, 6, and 7, respectively. There were no participants in H4 or H5. No differences between groups were found in nitrite concentration or systolic and diastolic BP before the intervention. The H1 group (all ancestral alleles) was the only group that presented a decrease in systolic and diastolic BP and an increase in nitrite concentration. Groups H2, H3, and H7 only presented a reduction in systolic BP, while group H6 presented a decrease in both systolic and diastolic BP. H7 also resulted in increased nitrite concentration. It is important to note that haplotypes that descriptively demonstrated trends towards the lowest magnitudes of SBP decrease, H6 and H7, presented two variant alleles. Multicomponent training did not affect the results of the H8 group.

Table 6 presents the haplotype analysis showing the effect of multicomponent training on oxidative stress and antioxidant activity. At baseline, all groups were different from the H3 group for malondialdehyde. All groups presented decreased MDA values after the intervention. For antioxidant activity, only the H1 group presented improvement in the two variables (total antioxidant capacity and glutathione). The H6 group increased only the total antioxidant capacity.

As in the isolated analysis of each polymorphism, the analysis grouped by haplotypes did not influence the effects of multicomponent training on physical fitness. The H1, H2, H3, H6, H7, and H8 presented similar improvements in the motor tests performed (data not shown) indicating that the multicomponent training was effective.

## 4. Discussion

### 4.1. General Data

The general results of this study demonstrated a positive effect of training on systolic and diastolic blood pressure, nitrite concentration, redox status, and physical fitness, independent of the genotype (Table 2). Multicomponent training was chosen for the intervention as physical changes related to the aging process affect various motor skills and abilities. In addition, multicomponent training includes the training of various capacities and motor skills (aerobic capacity, muscular strength, flexibility, coordination, agility, and balance) and is in accordance with ACSM guidelines [3]. This training protocol has been demonstrated to be more effective in promoting significant improvements in physical fitness than other investigated exercise protocols (aerobic, concurrent training, and strength) [6].

Physical training can increase eNOS activity and antioxidant activity and decrease values of BP [9, 14]. However, the influence of physical exercise on plasma nitrite concentration is still controversial [36]. Some studies demonstrate an increase in NO bioavailability and production in response to acute and chronic physical exercise [36, 39], while others indicate that plasma nitrate/nitrite levels are unchanged after long-term aerobic exercise training in older adults [16]. In the present investigation, it was possible to observe that 12 weeks of multicomponent training was effective for increasing nitrite concentration and lowering BP, besides improving oxidative stress, antioxidant activity, and physical fitness.

### 4.2. Genotype Analyses

The main finding of the genotype analysis was that in the blood pressure response, nitrite concentration and antioxidant activity seemed to be associated with the genotype. In the current study, it was possible to verify in the isolated analysis that the groups with the variant genotypes did not present improvement, or when they did present improvement, descriptively it was of a trend towards a lower magnitude (∆%) of decrease in systolic and diastolic BP and increase in nitrite concentration compared with the ancestral genotype groups [-786T>C, 894G>T (Glu298Asp), and intron 4b/a]. Only the groups with ancestral genotype in the three NOS3 polymorphisms studied showed improvement in glutathione, while the group with variant genotypes of intron 4b/a (4b4a+4a4a) was the only group that did not present improvement in total antioxidant capacity among all the studied groups; all groups improved malondialdehyde values. Few studies have been carried out seeking to evaluate similar aspects to the present study, and the findings are contradictory. Esposti et al. found similar improvements after eight weeks of aerobic exercise training in systolic and diastolic BP in all groups [with and without variants in -786T>C, 894G>T (Glu298Asp), and intron 4b/a]. However, they did not find improvement in malondialdehyde or nitrite/nitrate concentration [16]. Silva et al. investigated the individual and combined effects of three variants of the eNOS gene (-786T>C, Glu298Asp, and intron 4b/a) on vascular reactivity before and after exercise in male and female adults. The authors showed that participants with the Glu298Asp variant had lower vascular reactivity than wild counterparts [40]. These contradictory results can be explained by differences in the populations studied, ages of participants, analyses performed, and type of exercise program.

### 4.3. Haplotype Analysis

In the haplotype analysis, as in the genotype analysis, the response to multicomponent training in decreasing blood pressure or increasing nitrite concentration and antioxidant activity was also associated with the haplotypes. The H8 group, with the three variant alleles, showed improvement only in malondialdehyde and did not demonstrate an increase in nitrite concentration or antioxidant activity (total antioxidant capacity and glutathione) or a reduction in systolic and diastolic BP. However, the H1 group, carrying all three ancestral alleles, demonstrated improvement in nitrite concentration, glutathione and total antioxidant capacity, and a reduction in malondialdehyde and systolic and diastolic BP. The abovementioned study by Silva et al. regarding the combined impact of three variants in the eNOS showed that participants carrying Glu298Asp variants reduced the exercise-mediated increase in vascular reactivity, particularly when it occurred concomitantly with the -786T>C variants [40]. Zago et al. found reduced nitrite concentration in the group with eNOS genotype variants (-786: TC + CC and Glu298Asp: GluAsp + AspAsp); however, this group increased nitrite concentration and decreased BP, after eight weeks of aerobic exercise training [36]. Furthermore, Sponton et al. showed that the NOS3 polymorphism for intron 4b/a mitigated the beneficial effects of aerobic exercise training for systolic and diastolic BP but was not associated with nitrite/nitrate levels or redox state. Paradoxical responses were found for positions -T786T>C and G894G>T (Glu298Asp) for the NOS3 polymorphisms. Their data showed that two haplotype groups, H3 and H5, were unresponsive in lowering systolic and diastolic BP in response to exercise training in both office measurements and ambulatory BP monitoring [17].

Similarly to the results in the current study, Sponton et al. presented a similar response in groups H1 and H6, without any of the three variants and with two variants (-786T>C and Glu298Asp), respectively, showing improvement in systolic and diastolic BP and antioxidant activity [17]. However, in contrast, the current study also demonstrated improvement in nitrite concentrations after the intervention in groups H1 and H7 and improved levels of malondialdehyde in all groups.

Few studies have investigated the influence of NOS3 polymorphisms on the response to a physical training program [16, 17, 36]. While da Silva et al. [15] have already established an interaction of the three polymorphisms (haplotypes) with basal nitrite concentration values, Sponton et al. [17] investigated the response of physical training program in association with the NOS3 haplotypes.

The novelty of the current study is the use of a multicomponent program in the physical training intervention, and, besides the isolated analysis of each polymorphism, we performed the interaction analysis (haplotypes) among polymorphisms and analyzed the influence on the response to multicomponent training—as aforementioned, a previous study performed aerobic exercise. Our results showed that the presence of polymorphisms in the isolated analysis of each polymorphism affected the response of decreased systolic and diastolic BP and improved nitrite concentration and antioxidant activity. In the interaction (haplotypes) analysis, the group composed of all ancestral alleles (H1) was the only one to show improvement in all variables studied (systolic and diastolic BP, nitrite concentration, redox status, and physical fitness), while the group composed of all variant alleles (H8) only showed improvement in some variables of redox status and physical fitness. Therefore, preventive actions to change lifestyles for women's health are crucial since women live longer than men and effective prevention could decrease the high cost to the health care system for this population.

In conclusion, our data demonstrated few differences between the groups at baseline in both analyses, in each polymorphism alone and in the interaction between them (haplotypes). However, it was possible to observe a positive effect of training on the studied health parameters and the response to decreased blood pressure and improved nitrite concentration and antioxidant activity seemed to be associated with the genotype. Studies in this area are important as it could be possible in the future to consider genetic variants when choosing a more suitable exercise training program for each individual.

## Figures and Tables

**Figure 3:**
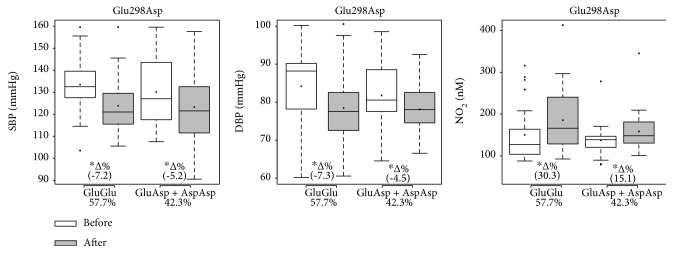
Effects of 12 weeks of multicomponent training on the systolic blood pressure (SBP), diastolic blood pressure (DBP), and nitrite concentration (NO_2_) of 52 older adult women with or without variant genotypes for the eNOS gene at position 894G>T (Glu298Asp). ^∗^*p* < 0.05 compared with before intervention (the same group). Linear mixed-effects models.

**Figure 4:**
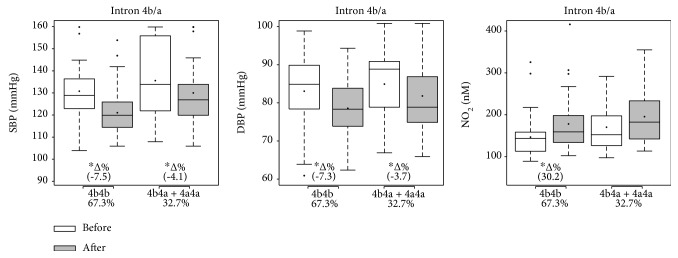
Effects of 12 weeks of multicomponent training on the systolic blood pressure (SBP), diastolic blood pressure (DBP), and nitrite concentration (NO_2_) of 52 older adult women with or without variant genotypes for the eNOS gene intron 4b/a. ^∗^*p* < 0.05 compared with before intervention (the same group). Linear mixed-effects models.

**Table 1:** Distribution of the genotypes of participants.

Genotypes	(*n* = 52)	%
-786T>C		
TT	25	48.1
TC	23	44.2
CC	4	7.7
894G>T (Glu298Asp)		
GluGlu	30	57.7
GluAsp	20	38.5
AspAsp	2	3.8
Intron 4b/a		
4b4b	35	67.3
4b4a	17	32.7
4a4a	0	0

**Table 2:** Effect of multicomponent training on blood pressure, nitrite concentration, oxidative stress, antioxidant activity, and physical fitness of 52 older adult women.

	Before	After	∆%
Age (years)	61.9 (8.7)		
SBP (mmHg)	132 (15)	124 (14)^∗^	−6.3
DBP (mmHg)	83 (9)	78 (8)^∗^	−6.1
NO_2_ (nM)	112 (55)	141 (69)^∗^	26.3
MDA (*μ*M)	4.9 (1.6)	2.6 (1.2)^∗^	−46.6
GSH (*μ*M)	3.5 (0.6)	3.6 (0.8)	2.8
TAC (*μ*M)	0.32 (0.11)	0.39 (0.12)^∗^	21.9
EFT (reps)	17.1 (3.3)	20.1 (4.1)^∗^	17.7
SS (reps)	14.4 (3.4)	17.8 (4.8)^∗^	23.1
6 min WT (m)	512 (62)	559 (60)^∗^	9.3

Data are reported as means (SD) before and after 12 weeks of multicomponent training for 52 women. SBP = systolic blood pressure; DBP = diastolic blood pressure; NO_2_ = nitrite concentration; MDA = malondialdehyde; GSH = glutathione; TAC = total antioxidant capacity; EFT = elbow flexion test; SS = sit and stand up; 6 min WT = six-minute walk test. ^∗^*p* < 0.05 compared with before intervention (the same group). Linear mixed-effects models.

**Table 3:** Effects of 12 weeks of multicomponent training on oxidative stress, antioxidant activity, and physical fitness of 52 older adult women with or without variant genotypes for the eNOS gene at position -786T>C.

	TT (25)			TC + CC (27)		
	Before	After	∆%	Before	After	∆%
Age (years)	63.7 (9)			60.7 (8.4)		
MDA (*μ*M)	5 (1.83)	2.7 (1.5)^∗^	−45.2	4.9 (1.4)	2.5 (0.8)^∗^	−47.7
GSH (*μ*M)	3.6 (0.5)	3.8 (0.9)^∗^	6.1	3.5 (0.7)	3.5 (0.8)	−0.7
TAC (*μ*M)	0.32 (0.11)	0.38 (0.11)^∗^	18.8	0.35 (0.12)	0.40 (0.12)^∗^	14.3
EFT (reps)	16.5 (3.1)	19.6 (3.6)^∗^	18.7	17.6 (3.4)	20.6 (4.5)^∗^	16.8
SS (reps)	14 (3.6)	18.1 (5.3)^∗^	28.8	14.8 (3.2)	17.5 (4.4)^∗^	18
6 min WT (m)	508 (70)	559 (75)^∗^	10.1	516 (56)	560 (43)^∗^	8.6

Data are reported as means (SD) before and after 12 weeks of multicomponent training for 52 women. MDA = malondialdehyde; GSH = glutathione; TAC = total antioxidant capacity; EFT = elbow flexion test; SS = sit and stand up; 6 min WT = six-minute walk test. ^∗^*p* < 0.05 compared with before intervention (the same group). Linear mixed-effects models.

**Table 4:** Effects of 12 weeks of multicomponent training on oxidative stress, antioxidant activity, and physical fitness of 52 older adult women with or without variant genotypes for the eNOS gene at position 894G>T (Glu298Asp).

	GluGlu (30)			GluAsp + AspAsp (22)		
	Before	After	∆%	Before	After	∆%
Age (years)	62 (8.4)			61.8 (9.2)		
MDA (*μ*M)	5 (1.7)	2.8 (1.4)^∗^	−43.1	4.8 (1.5)	2.3 (0.7)^∗^	−51.8
GSH (*μ*M)	3.6 (0.5)	3.7 (0.8)^∗^	4.8	3.5 (0.7)	3.5 (0.8)	0
TAC (*μ*M)	0.34 (0.12)	0.40 (0.12)^∗^	17.6	0.32 (0.08)	0.39 (0.12)^∗^	21.9
EFT (reps)	17.5 (3.1)	20.4 (4.2)^∗^	16.8	16.6 (3.5)	19.7 (3.9)^∗^	18.9
SS (reps)	15.3 (3.6)	18.6 (4.9)^∗^	21.5	13.2 (2.7)	16.6 (4.5)^∗^	25.4
6 min WT (m)	515 (66)	562 (67)^∗^	9.1	507 (58)	556 (49)^∗^	9.6

Data are reported as means (SD) before and after 12 weeks of multicomponent training for 52 women. MDA = malondialdehyde; GSH = glutathione; TAC = total antioxidant capacity; EFT = elbow flexion test; SS = sit and stand up; 6 min WT = six-minute walk test. ^∗^*p* < 0.05 compared with before intervention (the same group). Linear mixed-effects models.

**Table 5:** Effects of 12 weeks of multicomponent training on oxidative stress, antioxidant activity, and physical fitness of 52 older adult women with or without variant genotypes for the eNOS gene intron 4b/a.

	4b4b (35)			4b4a + 4a4a (17)		
	Before	After	∆%	Before	Before	∆%
Age (years)	61.7 (8.8)			62.1 (8.7)		
MDA (*μ*M)	4.9 (1.5)	2.4 (0.8)^∗^	−50.8	4.9 (1.9)	3.1 (1.7)^∗^	−38.2
GSH (*μ*M)	3.6 (0.6)	3.8 (0.9)^∗^	5.3	3.4 (0.5)	3.3 (0.5)	−2.4
TAC (*μ*M)	0.31 (0.09)	0.40 (0.13)^∗^	29	0.34 (0.14)	0.38 (0.10)	11.8
EFT (reps)	16.7 (3)	19.8 (3.6)^∗^	18.1	17.8 (3.7)	20.8 (4.9)^∗^	16.9
SS (reps)	13.9 (3.2)	17.7 (5)^∗^	27.4	15.6 (3.4)	17.9 (4.4)^∗^	15.1
6 min WT (m)	511 (66)	558 (63)^∗^	9.3	514 (57)	563 (55)^∗^	9.4

Data are reported as means (SD) before and after 12 weeks of multicomponent training for 52 women. MDA = malondialdehyde; GSH = glutathione; TAC = total antioxidant capacity; EFT = elbow flexion test; SS = sit and stand up; 6 min WT = six-minute walk test. ^∗^*p* < 0.05 compared with before intervention (the same group). Linear mixed-effects models.

**Table 6:** Effects of 12 weeks of multicomponent training on oxidative stress and antioxidant activity of 52 older adult women grouped by haplotype (H1–H8).

	Haplotypes			MDA (*μ*M) before	MDA (*μ*M) after	∆**%**	GSH (*μ*M) before	GSH (*μ*M) after	∆%	TAC (*μ*M) before	TAC (*μ*M) after	∆%
	-786T>C	Intron 4	Glu298 Asp									
H1	T	4b	Glu	4.8 (1.5)^a^	2.5 (0.8)^∗^	−78.8	3.6 (0.5)	3.9 (1)^∗^	8.3	0.33 (0.10)	0.40 (0.12)^∗^	21.2
H2	T	4b	Asp	4 (1.2)^a^	1.9 (0.5)^∗^	−53.4	3.7 (0.4)	3.8 (0.8)	1.3	0.30 (0.05)	0.35 (0.02)	16.7
H3	T	4a	Glu	7.3 (3.2)	5.5 (2.7)^∗^	−24.4	3.6 (0.7)	3.6 (0.7)	0	0.33 (0.19)	0.36 (0.15)	9.1
H4	T	4a	Asp	—	—	—	—	—	—	—	—	—
H5	C	4b	Glu	—	—	—	—	—	—	—	—	—
H6	C	4b	Asp	5.3 (1.6)^a^	2.5 (0.8)^∗^	−52.7	3.6 (0.8)	3.7 (0.9)	1.9	0.33 (0.09)	0.42 (0.15)^∗^	27.3
H7	C	4a	Glu	4.5 (1.1)^a^	2.7 (1.1)^∗^	−40.7	3.4 (0.4)	3.4 (0.6)	−0.3	0.37 (0.14)	0.41 (0.11)	10.8
H8	C	4a	Asp	4.3 (1.2)^a^	2.2 (0.3)^∗^	−47.4	3.2 (0.4)	3 (0.2)	−8.1	0.29 (0.10)	0.35 (0.08)	20.7

Data are reported as means (SD) before and after 12 weeks of multicomponent training for 52 women. MDA = malondialdehyde; GSH = glutathione; TAC = total antioxidant capacity. ^∗^*p* < 0.05 compared with before intervention (the same group). ^a^*p* < 0.05 versus H3 before intervention. Linear mixed-effects models.

## References

[1] UNFPA – United Nations Population Fund (2012). *Ageing in the twenty-First Century: A Celebration and a Challenge*.

[2] Rattan S. I. S. (2014). Aging is not a disease: implications for intervention. *Aging and Disease*.

[3] American College of Sports Medicine, Chodzko-Zajko W. J., Proctor D. N. (2009). American College of Sports Medicine position stand: exercise and physical activity for older adults. *Medicine and Science in Sports and Exercise*.

[4] Malachias M. V. B., de Souza W. K. S. B., Plavnik F. L. (2016). VII Diretriz Brasileira de Hipertensão Arterial. *Arquivos Brasileiros de Cardiologia*.

[5] North B. J., Sinclair D. A. (2012). The intersection between aging and cardiovascular disease. *Circulation Research*.

[6] Neves L. M., Diniz T. A., Rossi F. E. (2016). The effect of different training modalities on physical fitness in women over 50 year of age. *Motriz*.

[7] Garber C. E., Blissmer B., Deschenes M. R. (2011). Quantity and quality of exercise for developing and maintaining cardiorespiratory, musculoskeletal, and neuromotor fitness in apparently healthy adults: guidance for prescribing exercise. *Medicine and Science in Sports and Exercise*.

[8] Parati G., Ochoa J. E., Lombardi C., Bilo G. (2013). Assessment and management of blood-pressure variability. *Nature Reviews Cardiology*.

[9] Jacomini A. M., de Souza H. C., Dias Dda S. (2016). Training status as a marker of the relationship between nitric oxide, oxidative stress, and blood pressure in older adult women. *Oxidative Medicine and Cellular Longevity*.

[10] Modun D., Giustarini D., Tsikas D. (2014). Nitric oxide-related oxidative stress and redox status in health and disease. *Oxidative Medicine and Cellular Longevity*.

[11] Moncada S., Higgs E. A. (2006). The discovery of nitric oxide and its role in vascular biology. *British Journal of Pharmacology*.

[12] Rush J. W. E., Green H. J., MacLean D. A., Code L. M. (2005). Oxidative stress and nitric oxide synthase in skeletal muscles of rats with post-infarction, compensated chronic heart failure. *Acta Physiologica Scandinavica*.

[13] Vanhoutte P. M. (2003). Endothelial control of vasomotor function – from health to coronary disease. *Circulation Journal*.

[14] Trape A. A., Jacomini A. M., Muniz J. J. (2013). The relationship between training status, blood pressure and uric acid in adults and elderly. *BMC Cardiovascular Disorders*.

[15] da Silva R. F., Sertório J. T., Lacchini R. (2014). Influence of training status and eNOS haplotypes on plasma nitrite concentration in normotensive older adults: a hypothesis-generating study. *Aging Clinical and Experimental Research*.

[16] Esposti R. D., Sponton C. H. G., Malagrino P. A. (2011). Influence of eNOS gene polymorphism on cardiometabolic parameters in response to physical training in postmenopausal women. *Brazilian Journal of Medical and Biological Research*.

[17] Sponton C. H., Esposti R., Rodovalho C. M. (2014). The presence of the NOS3 gene polymorphism for intron 4 mitigates the beneficial effects of exercise training on ambulatory blood pressure monitoring in adults. *American Journal of Physiology Heart and Circulatory Physiology*.

[18] Bouchard C., Antunes-Correa L. M., Ashley E. A. (2015). Personalized preventive medicine: genetics and the response to regular exercise in preventive interventions. *Progress in Cardiovascular Diseases*.

[19] Karasik D., Newman A. (2015). Models to explore genetics of human aging. *Longevity Genes – Advances in Experimental Medicine and Biology*.

[20] Casas J. P., Cavalleri G. L., Bautista L. E., Smeeth L., Humphries S. E., Hingorani A. D. (2006). Endothelial nitric oxide synthase gene polymorphisms and cardiovascular disease: a HuGE review. *American Journal of Epidemiology*.

[21] Pereira T. V., Rudnick M., Cheung B. M. (2007). Three endothelial nitric oxide (NOS3) gene polymorphisms in hypertensive and normotensive individuals: meta-analysis of 53 studies reveals evidence of publication bias. *Journal of Hypertension*.

[22] Zintzaras E., Kitsios G., Stefanidis I. (2006). Endothelial NO synthase gene polymorphisms and hypertension: a meta-analysis. *Hypertension*.

[23] Borg G., Noble B. J., Wilmore J. H. (1954). Perceived exertion. *Exercise and Sport Sciences Reviews*.

[24] Matsudo S., Araujo T., Matsudo V. (2001). Questionário Internacional de Atividade Física (I-PAQ): estudo de validade e reprodutibilidade no Brasil. *Revista Atividade Física & Saúde*.

[25] Brasil. Ministério da Saúde. Secretaria de Atenção à Saúde. Departamento de Atenção Básica (2008). *Protocolos do Sistema de Vigilância Alimentar e Nutricional - SISVAN/Ministério da Saúde, Secretaria de Atenção à Saúde*.

[26] Abep. Associação Brasileira de Empresas de Pesquisa (2008). *Critério de classificação econômica Brasil*.

[27] Rikli R. E., Jones J. C. (2008). *Teste de aptidão física para idosos*.

[28] Pinheiro L. C., Montenegro M. F., Amaral J. H., Ferreira G. C., Oliveira A. M., Tanus-Santos J. E. (2012). Increase in gastric pH reduces hypotensive effect of oral sodium nitrite in rats. *Free Radical Biology & Medicine*.

[29] Fan Q., Chen L., Cheng S. (2014). Aging aggravates nitrate-mediated ROS/RNS changes. *Oxidative Medicine and Cellular Longevity*.

[30] Gerard-Monnier D., Erdelmeier I., Regnard K., Moze-Henry N., Yadan J. C., Chaudiere J. (1998). Reactions of 1-methyl-2-phenylindole with malondialdehyde and 4-hydroxyalkenals. Analytical applications to a colorimetric assay of lipid peroxidation. *Chemical Research in Toxicology*.

[31] Ekstrom T., Garberg P., Egestad B., Hugberg J. (1988). Recovery of malondialdehyde in urine as a 2,4-dinitropjenylhydrazine derivate analyzed with high-performance liquid chromatography. *Chemico-Biological Interactions*.

[32] Erel O. (2004). A novel automated direct measurement method for total antioxidant capacity using a new generation, more stable ABTS radical cation. *Clinical Biochemistry*.

[33] Costa C. M., Santos R. C. C., Lima E. S. (2006). A simple automated procedure for thiol measurement in human serum samples. *Jornal Brasileiro de Patologia e Medicina Laboratorial*.

[34] Lahiri D. K., Nurnberger J. I. (1991). A rapid non-enzymatic method for the preparation of HMW DNA from blood for RFLP studies. *Nucleic Acids Research*.

[35] Vasconcellos V., Lacchini R., Jacob-Ferreira A. L. (2010). Endothelial nitric oxide synthase haplotypes associated with hypertension do not predispose to cardiac hypertrophy. *DNA and Cell Biology*.

[36] Zago A. S., Park J. Y., Fenty-Stewart N., Kokubun E., Brown M. D. (2010). Effects of aerobic exercise on the blood pressure, oxidative stress and eNOS gene polymorphism in pre-hypertensive older people. *European Journal Applied Physiology*.

[37] Marroni A. S., Metzger I. F., Souza-Costa D. C. (2005). Consistent interethnic differences in the distribution of clinically relevant endothelial nitric oxide synthase genetic polymorphisms. *Nitric Oxide*.

[38] Schall R. (1991). Estimation in generalized linear models with random effects. *Biometrika*.

[39] Santana H. A., Moreira S. R., Asano R. Y. (2013). Exercise intensity modulates nitric oxide and blood pressure responses in hypertensive older women. *Aging Clinical and Experimental Research*.

[40] Silva B. M., Neves F. J., Rocha N. G. (2013). Endothelial nitric oxide gene haplotype reduces the effect of a single bout of exercise on the vascular reactivity in healthy subjects. *Translational Research*.

